# Apparent absence of *Giardia* infections among children under 5-years of age with acute watery diarrhoea in Abakaliki, Nigeria

**DOI:** 10.1017/S0950268818003151

**Published:** 2018-12-03

**Authors:** A. M. Efunshile, O. Ezeanosike, O. N. I. Onyekachi, M. I. Ugwu, B. König, L. J. Robertson

**Affiliations:** 1 Department of Medical Microbiology, Ebonyi State University, Abakaliki, Nigeria; 2 Department of Medical Microbiology, Federal Teaching Hospital, Abakaliki, Nigeria; 3 Department of Paediatrics, Federal Teaching Hospital, Abakaliki, Nigeria; 4 Department of Paediatrics, Ebonyi State University, Abakaliki, Nigeria; 5 Institute of Medical Microbiology and Epidemiology of Infectious Diseases, University Teaching Hospital, Leipzig, Germany; 6 Parasitology, Department of Food Safety and Infection Biology, Faculty of Veterinary Medicine, Norwegian University of Life Sciences, PO Box 369 Sentrum, 0102 Oslo, Norway

**Keywords:** *Cryptosporidium*, diarrhoea, *Giardia duodenalis*, parasitic disease epidemiology and control

## Abstract

Although the impact of diarrhoeal disease on paediatric health in Nigeria has decreased in recent years, it remains an important cause of morbidity and mortality in children under 5 years. *Rotavirus* is recognised as an important aetiological agent, but information on the contribution of intestinal protozoa to watery diarrhoea in this age group in Nigeria is scarce. In this cross-sectional study, faecal samples from children admitted to healthcare centres in Abakaliki, Nigeria with acute watery diarrhoea (*N* = 199) and faecal samples from age-matched controls (*N* = 37) were examined for *Cryptosporidium* and *Giardia* using immunofluorescent antibody testing and molecular methods. *Cryptosporidium* was identified in 13 case samples (6.5%) and no control samples. For three samples, molecular characterisation indicated *C. hominis*, GP60 subtypes IaA30R3, IaA14R3 and IdA11. *Giardia* was not detected in any samples. This contrast in prevalence between the two intestinal protozoa may reflect their variable epidemiologies and probably differing routes of infection. Given that these two parasitic infections are often bracketed together, it is key to realise that they not only have differing clinical spectra but also that the importance of each parasite is not the same in different age groups and/or settings.

## Introduction

In Nigeria, deaths from diarrhoea among children below the age of 5 years decreased by just over 20% between 2005 and 2015, but nevertheless remained substantial at 327.6 per 100 000 children; in contrast, the global mortality from diarrhoea in children in this age group is estimated at 74.3 per 100 000 [1]. Among the aetiologies associated with mortality due to diarrhoea in Nigeria in children under 5 years, rotaviral enteritis had the highest impact (45%), with cryptosporidiosis considered responsible for 14.3% [1]. In the global enteric multicentre study (GEMS) in which the under-5 years age group was stratified into three age groups (under 11 months, 12–23 months, and 24–59 months), it was noticed that at most study sites included, *Cryptosporidium* tended to be unimportant as a diarrhoeal pathogen in the oldest age stratum, with the greatest burden found in the youngest age groups [2]. An extended analysis has shown that: (1) diarrhoeal diseases in general in children under 5-years has a greater impact regarding long-term health burden than previously estimated [3], and (2) the considerable short-term impact of acute cryptosporidiosis on morbidity in this age group underestimates the true burden by 153% [4].

Another intestinal protozoan parasite that is often considered together with *Cryptosporidium*, is *Giardia duodenalis*. Although infection with *Giardia* is known to cause diarrhoea, its role in childhood diarrhoea is less certain and it tends not to be associated with increased mortality. One study estimated that in the World Health Organization AFRO region (which includes Nigeria), in the under 5-years age group, around 4-times more cases of diarrhoea were due to *Giardia* infection than due to *Cryptosporidium* infection [5]. However, the GEMS study found that there was no significant association between *Giardia* and symptoms of moderate-to-severe diarrhoea in the cohorts of children under 5-years included in the study and, indeed, in univariate analyses of the oldest age stratum (12–59 months) there was a significantly higher probability of identifying *Giardia* in controls than in patients in most of the study sites, including those in Africa [2]. A previous meta-analysis of published data had also concluded that although *Giardia* is associated with persistent diarrhoea in children in developing countries, it does not generally cause acute paediatric diarrhoea among infants and children in those countries [6]. A follow-up study of Israeli-Arab children aged 2.3–4.7 years indicated that infection with *Giardia* may even lower the risk of acute diarrhoea within this age group [7].

Our study provides further data on the role of both *Cryptosporidium* and *Giardia* in paediatric diarrhoea in Nigeria. Furthermore, efforts were made to identify any risk factors for infection with either of these parasites and information regarding symptoms and any home treatment prior to admission was also collected.

## Methods

### Study design and setting

This was a cross-sectional study carried out at the two large healthcare centres within Abakaliki, Nigeria. Abakaliki is the capital city of Ebonyi State, SE Nigeria, approximately 650 km east of Lagos; in 2006 the population of Abakaliki was around 152 000 persons.

Stool samples were collected between the months of December 2016 and March 2017, which coincided with the dry season of the year. Caregivers (parents/guardians) of all the diarrhoeic children fulfilling the inclusion criteria for the study and admitted to the healthcare centres during this period were invited to have their children enrolled in our study. Participation was initiated on receipt of written, informed consent from the caregivers. A Strobe checklist was completed for this study and is included as Supplementary Material, which is available via the Cambridge Core website.

### Study population

Children included in the study were 5-years-old or younger and had been diagnosed with acute watery diarrhoea by the managing paediatricians. Children co-infected with malaria, respiratory tract infections, or other disease conditions were excluded.

### Ethical considerations

Approval for the study was given by the Review and Ethics Committee of the Federal Teaching Hospital, Abakaliki. An informed consent form was signed by parents/guardians prior to enrolment in the study; information in the form made clear that participation in the study was voluntary and participants could opt out of the study at any time without prejudice to the quality of treatment received by their children. Questionnaires designed for data collection were coded to be anonymous such that it was not possible to identify individual patients. Information was collected using the language that the caregivers felt most comfortable using.

### Questionnaire

Data collected by questionnaire include sociodemographic details as well as information regarding the particular episode of diarrhoea.

### Sample collection and analysis

In addition to routine diagnostics conducted at the healthcare centres, stool samples were collected from 200 cases and 37 age-matched controls (children attending the routine immunization clinics at the same healthcare centres treating the diarrhoea case-patients, with no diarrhoea and apparently healthy; all control children were 5-years or younger, with over 50% younger than 11-months old). Samples were preserved in absolute ethanol and transported to the Parasitology Laboratory of the Norwegian University of Life Sciences. Here, each sample was homogenised, washed twice in laboratory-grade water and concentrated by centrifugation. Detection of *Cryptosporidium* oocysts and *Giardia* cysts was conducted by standard immunofluorescent antibody test (IFAT) on 10 µl subsamples that were air-dried, methanol-fixed and stained with FITC-labelled monoclonal antibody (Mab: Aqua-glo, Waterborne Inc., New Orleans, USA) and 4′,6-diamidino-2-phenylindole (DAPI). Prepared slides were examined by fluorescence microscopy, using the appropriate filters, at magnifications of 200 × and 400 ×  and *Cryptosporidium* oocysts and/or *Giardia* cysts enumerated and DAPI inclusion recorded.

DNA was extracted from all those samples that were positive for *Cryptosporidium* oocysts and every fourth of the other samples (both cases and controls; a total of 59 cases samples and nine control samples) by resuspending 50% of the remaining pellet in 100 µl Tris–EDTA buffer and heating for 1 h in a heat block set at 100 °C. DNA was then isolated using a QIAmp DNA mini-kit (QIAGEN GmbH, Germany) following the manufacturer's protocol.

For those samples that were positive for *Cryptosporidium*, fragments of two genes were used for molecular investigations; these were the SSU rRNA gene (approximately 800 bp) and the GP60 gene (approximately 950 bp;), using published primers and protocols [8, 9]. For all samples from which DNA had been extracted, PCR with primers targeting the SSU rRNA gene of *Giardia* was also conducted using published primers and protocols [10]. Each set of PCR included a negative (laboratory-grade water) control and a positive control.

PCR amplification products from positive samples were purified (High Pure PCR product purification kit, Roche Applied Science) according to the manufacturer's protocol and sequenced on both strands at a commercial facility (Macrogen, South Korea). Chromatograms were examined and sequences adjusted manually. Sequence searches were conducted using BLAST (http://blast.ncbi.nlm.nih.gov/Blast.cgi) and, in addition, for GP60 sequences, the 5′ end was manually checked and tandem repeats of the serine-coding trinucleotides enumerated to determine subtype family.

Analysis of the samples for viral pathogens was conducted at the Institute of Virology, University of Leipzig, Germany using multiplex real-time polymerase chain reaction (PCR) standard protocol; the detailed data about viruses are intended for separate presentation.

### Data handling and statistical analysis

Data were compiled in an excel database; and then exported to SPSS version 24 for analysis. Associations were investigated by contingency table analysis (Fisher's Exact Test).

## Results

### Study population, symptoms and case management

Of the 200 case samples collected, one was not suitable for analysis due to specimen leakage; hence 199 cases and 37 controls were included in our final analysis.

The majority of the study participants were males (60.8%) and within the age range of 0–11-months-old (68.4%). Most participants came from rural areas and were not exclusively breastfed (72.0%), (Table 1).

**Table 1.** Descriptive characteristics of cases and potential risk factors for infection

Parameter	Number (%)
Age group	
0–11 months	136 (68.4)
12–24 months	56 (28.1)
25–60 months	7 (3.5)
Gender	
Male	121 (60.8)
Female	78 (39.2)
Highest level of maternal education	
No formal education	52 (26.1)
Primary School	9 (4.5)
Secondary school	108 (54.3)
Tertiary level	30 (15.1)
Place of residence	
Rural	111 (55.8)
Urban	88 (44.2)
Major source of drinking water	
Factory packaged water	155 (77.9)
Borehole	37 (18.7)
Rain	3 (1.5)
Stream	2 (1.0)
Well	2 (1.0)
Type of toilet facility	
Water closet	128 (64.3)
Pit latrine	33 (16.6)
Bush	38 (19.1)
Exclusively breastfed	
Yes	56 (28.1)
No	144 (71.9)

In most of the children, the diarrhoea was associated with fever (98.7%) and vomiting (92.5%), and although mucus in faeces was common, only 5% passed blood-stained stools (Table 2).

**Table 2.** Clinical features associated with the diarrhoeal cases

Clinical feature	Number (%)
Fever	196 (98.5)
Vomiting	184 (92.5)
Mucus in stool	140 (70.4)
Blood in stool	10 (5.0)
Diarrhoeal persistence for over 1 week	52 (26.1)

In the children's homes, oral rehydration solutions were commonly used (98.0%), whereas the use of zinc tablets (98%), intravenous fluids (92.5%) and antibiotics (87.0%) were the commonest hospital management practices (Table 3).

**Table 3.** Case management practices used for paediatric acute watery diarrhoea at home and in the hospitals

Case management practices	Number (%)
At home	
Continuous feeding	118 (59.3)
Non-prescription anti-diarrhoea drugs	31 (15.6)
Oral rehydration solutions	195 (98.0)
In hospital	
Intravenous fluid	184 (92.5)
Antibiotics	174 (87.4)
Probiotics	73 (36.7)
Zinc tablets	194 (98.0)
Vitamin A	163 (81.9)

### Aetiology of diarrhoea and occurrence of *Cryptosporidium* and *Giardia*

We did not detect *Giardia* cysts by IFAT in any of the stool samples, cases or controls and PCR directed towards the *Giardia* SSU rRNA gene were also negative for the nine control samples and 59 case samples tested. Thus, the prevalence of *Giardia* infection in this cohort was considered to be 0.00% (95% Confidence Intervals (CI) 0.00–1.89).

We did not detect *Cryptosporidium* oocysts in any of the control samples, but oocysts were identified in 13 of the 199 stool samples from cases (6.5%; 95% CI 3.86–10.85). Of these positive samples, five contained low numbers of oocysts (20–30 oocysts in the whole slide), seven had moderate numbers of oocysts (up to five oocysts per field of view) but five of these exhibited poor or absent DAPI staining and one sample had very high numbers of oocysts. The number of oocysts per gram of faecal sample could not be estimated due to lack of information on the quantity of stool sample preserved in ethanol.

Of the 13 *Cryptosporidium*-positive samples, for all of which molecular characterisation was attempted, sequences were obtained only for three (the sample with high numbers of oocysts and the two samples with moderate numbers of oocysts and good DAPI staining). All three were *Cryptosporidium hominis*, two were of GP60 subtype family Ia (IaA30R3 and IaA14R3) and one of GP60 subtype Id (IdA11).

No associations between *Cryptosporidium* infection and other variables (age, gender, rural or urban residence) were identified.

*Rotavirus* was found in 92.3% of the cases; other agents detected included *Enterovirus* (20.8%), *Astrovirus* (8.6%), *Parechovirus* (8.1%) and *Sapovirus* (2.7%). *Ascaris* eggs and *Entamoeba* spp. cysts were also seen in 0.5% (1/199) and 1.0% (2/199) of cases, respectively.

## Discussion

The results reported here support those of earlier studies that suggest that in countries such as Nigeria, *Giardia* infection is not associated with paediatric diarrhoea. Indeed, a meta-analysis of case-control and cohort studies has indicated that there is a significant inverse association between acute diarrhoea and *Giardia* in stools in children in non-industrialised settings [6]. Thus, these studies have also often shown that control subjects (without diarrhoea) are more frequently infected with *Giardia*, suggesting that *Giardia* may mediate a protective effect (e.g. [7, 11–14]), but this was not seen in our study. The nature of the proposed protective effect suggested from other studies is unclear, although changes in mucosal immunity or suppression of other enteric pathogens have been suggested; it is worth noting that a study from Tanzania found that the apparently protective effect of *Giardia* infection in children of under 6-years was abrogated when multi-nutrient supplementation was used [15]. An alternative possibility is that in profuse watery diarrhoea caused by any aetiology (bacteriological, viral, parasitological), any resident *Giardia* trophozoites might be ‘washed out’ before they have time to encyst, hence the lack of *Giardia* cysts in the samples of diarrhoeic patients, including in our study. It could then be argued that *Giardia* DNA could have been detected; however, as the samples were collected from children attending healthcare centres due to diarrhoeal disease, it may be likely that any resident *Giardia* are washed out in early bouts of diarrhoea prior to admission to the healthcare centre and collection of the faecal sample. Such a situation could also, perhaps, explain why previous studies [7, 11–14] have suggested that *Giardia* infection may exert a protective effect.

Thus, the apparent lack of any *Giardia*-positive samples in this cohort (cases and controls) may not be as surprising as first appears. Indeed, giardiasis itself is associated with fatty, rather than watery diarrhoea. Preservation in ethanol has been recommended for faecal samples possibly containing *Giardia* cysts, for subsequent detection by either microscopy (IFAT) or PCR [16]. Nevertheless, degradation during transport cannot be completely excluded as the reason for the lack of detection of *Giardia*, either cyst structures detected by IFAT, or DNA that can be amplified by targeted PCR. However, this seems unlikely given the detection of *Cryptosporidium* in some samples. Indeed, previous studies from Nigeria investigating infections in children under 5-years of age have tended to detect *Giardia* cases at a relatively low prevalences (e.g. one *Giardia*-positive 6-month-old with enteritis among six children below 2 years [17]; one child (below 4-years of age) among 215 children with diarrhoea and no *Giardia* infections among 100 age-matched controls without diarrhoea [18]). However, investigations amongst older children (school-age children, 7–17 years) have tended to show a higher prevalence of *Giardia* infection, e.g., 12.3% [19] and 37.2% [20]; in neither of these studies from school-age children was diarrhoea reported among the subjects.

The 6.5% prevalence of *Cryptosporidium* infection in our study was within the range reported by other studies from Nigeria in children aged below 5 years. For example, a 4.8% prevalence among 165 children below 5-years of age with diarrhoea was found in Jos, with *C. hominis* the predominant species, as we found, and subtype Id [21]; a 5.6% prevalence of *Cryptosporidium* was reported in children with diarrhoea below 6-years of age from Ebonyi state, with both *C. hominis* and *C. parvum* identified and, among the *C. hominis* isolates, four different GP60 subtypes, including two in the Ia family [22]; a prevalence of *Cryptosporidium* infection was found in 11.1% of 21 diarrhoeic children less than 12 months of age from Oyo state; two of the isolates were *C. hominis*, both subtype IaA24R3 [23].

Our data support the higher occurrence of *C. hominis* species in Africa, despite just under 50% of the infected children (6 of 13) living in rural areas, where *C. parvum* may be more common due to associations with animal infections. However, the data provide no information regarding likely vehicles for infection and, given the low number of isolates characterised using molecular methods, our data should be treated with caution regarding extrapolation to other settings.

In conclusion, our results demonstrate that although viral infections appear to be of greatest importance in paediatric diarrhoea in this setting, *Cryptosporidium* infection is also of relevance. However, *Giardia* infection seems to be of limited importance in this age group of children, both for asymptomatic children and those with watery diarrhoea. Given that *Giardia* infections occur relatively frequently in older children, we speculate that our data probably reflect the differing epidemiologies and potentially differing transmission routes for these two parasites. This is important, as many studies tend to consider both *Cryptosporidium* and *Giardia* as being very similar epidemiologically, probably due to the fact that they are both intestinal protozoan parasites (albeit of widely differing phylogeny and biology) and have both been associated with waterborne outbreaks.

## Supplementary material

For supplementary material accompanying this paper visit https://doi.org/10.1017/S0950268818003151. click here to view supplementary material
