# Supplementary material for: Apparent absence of Giardia infections among children under 5-years of age with acute watery diarrhoea in Abakaliki, Nigeria
Source: Epidemiol Infect. 2018 Dec 3;147:e58. doi: 10.1017/S0950268818003151 (PMC6518545; doi:10.1017/S0950268818003151)
Supplement: Supplementary file 1 [file S0950268818003151sup001.doc]

STROBE Statement—Checklist of items that should be included in reports of ***cross-sectional studies***

|  | Item No | Recommendation |
| --- | --- | --- |
| **Title and abstract** | 1 | (*a*) Indicate the study’s design with a commonly used term in the title or the abstract  CROSS-SECTIONAL IN ABSTRACT |
|  |  | (*b*) Provide in the abstract an informative and balanced summary of what was done and what was found  COMPLETED |
| Introduction | | |
| Background/rationale | 2 | Explain the scientific background and rationale for the investigation being reported  IN INTRODUCTION |
| Objectives | 3 | State specific objectives, including any prespecified hypotheses  LAST PARAGRAPH OF INTRODUCTION |
| Methods | | |
| Study design | 4 | Present key elements of study design early in the paper  FIRST PARAGRAPH OF MATERIALS AND METHODS |
| Setting | 5 | Describe the setting, locations, and relevant dates, including periods of recruitment, exposure, follow-up, and data collection  FIRST PARAGRAPH OF MATERIALS AND METHODS |
| Participants | 6 | (*a*) Give the eligibility criteria, and the sources and methods of selection of participants  SECOND PARAGRAPH OF MATERIALS AND METHODS |
| Variables | 7 | Clearly define all outcomes, exposures, predictors, potential confounders, and effect modifiers. Give diagnostic criteria, if applicable  MAIN OUTCOME WAS DETECTION (OR NOT) OF CRYPTOSPORIDIUM AND/OR GIARDIA |
| Data sources/ measurement | 8* | For each variable of interest, give sources of data and details of methods of assessment (measurement). Describe comparability of assessment methods if there is more than one group  METHODS SAME FOR SAMPLES FROM CHILDREN ADMITTED WITH DIARRHOEA AND NON-DIARRHOEIC CONTROLS |
| Bias | 9 | Describe any efforts to address potential sources of bias  NO BIAS IDENTIFIED |
| Study size | 10 | Explain how the study size was arrived at  BASED ON ENROLMENT OF CASES WITHIN THE TIME FRAME |
| Quantitative variables | 11 | Explain how quantitative variables were handled in the analyses. If applicable, describe which groupings were chosen and why |
| Statistical methods | 12 | (*a*) Describe all statistical methods, including those used to control for confounding  DATA HANDLING SECTION OF METHODS AND RESULTS |
|  |  | (*b*) Describe any methods used to examine subgroups and interactions  DATA HANDLING SECTION OF METHODS AND RESULTS |
|  |  | (*c*) Explain how missing data were addressed  PATIENT WITH INSUFFICIENT SAMPLE EXCLUDED FROM A |
|  |  | (*d*) If applicable, describe analytical methods taking account of sampling strategy  NOT APPLICABLE |
|  |  | (*e*) Describe any sensitivity analyses  NOT APPLICABLE |
| Results | | |
| Participants | 13* | (a) Report numbers of individuals at each stage of study—eg numbers potentially eligible, examined for eligibility, confirmed eligible, included in the study, completing follow-up, and analysed  SAMPLE COLLECTION SECTION, RESLTS, AND TABLE 1 |
|  |  | (b) Give reasons for non-participation at each stage  FIRST SECTION OF RESULTS |
|  |  | (c) Consider use of a flow diagram  DEEMED UNNECESSARY |
| Descriptive data | 14* | (a) Give characteristics of study participants (eg demographic, clinical, social) and information on exposures and potential confounders  FIRST SECTION OF RESULTS AND TABLE 1 |
|  |  | (b) Indicate number of participants with missing data for each variable of interest  NOT APPLICABLE |
| Outcome data | 15* | Report numbers of outcome events or summary measures  INCLUDED IN RESULTS TABLE OR TEXT |
| Main results | 16 | (*a*) Give unadjusted estimates and, if applicable, confounder-adjusted estimates and their precision (eg, 95% confidence interval). Make clear which confounders were adjusted for and why they were included  RESULTS TABLE OR TEXT |
|  |  | (*b*) Report category boundaries when continuous variables were categorized  OOCYST NUMBER CATEGORIES DESCRIBED |
|  |  | (*c*) If relevant, consider translating estimates of relative risk into absolute risk for a meaningful time period  NOT RELEVANT |
| Other analyses | 17 | Report other analyses done—eg analyses of subgroups and interactions, and sensitivity analyses  NOT RELEVANT |
| Discussion | | |
| Key results | 18 | Summarise key results with reference to study objectives  INCLUDED |
| Limitations | 19 | Discuss limitations of the study, taking into account sources of potential bias or imprecision. Discuss both direction and magnitude of any potential bias  LIMITATIONS REGARDING POSSIBLE DNA DEGRADATION DURING TRANSPORT NOTED AND THAT FEW CRYPTOSPORIDIUM ISOLATES WERE CHARACTERISED |
| Interpretation | 20 | Give a cautious overall interpretation of results considering objectives, limitations, multiplicity of analyses, results from similar studies, and other relevant evidence  THROUGHOUT DISCUSSION SECTION |
| Generalisability | 21 | Discuss the generalisability (external validity) of the study results  DISCUSSED THAT EXTRAPOLATION OF CRYPTOSPORIDIUM FINDINGS SHOULD BE UNDERTAKEN WITH CAUTION |
| Other information | | |
| Funding | 22 | Give the source of funding and the role of the funders for the present study and, if applicable, for the original study on which the present article is based  ABSENCE OF FUNDING INCLUDED IN SUBMISSION FORM |

*Give information separately for exposed and unexposed groups.
